# Gamified human resource management as a driver of employee engagement through intrinsic motivation

**DOI:** 10.3389/fpsyg.2025.1746973

**Published:** 2026-01-21

**Authors:** Xuming Zhang, Jinliang Wang, Yiming Zhu, Zhaoqiao Ding

**Affiliations:** 1 Guangdong University of Science and Technology, Dongguan, China; 2 Guangdong Vocational Institute of Public Administration, Guangdong, China; 3 Zhongshan Torch Polytechnic, Zhongshan, China; 4 City University of Macau, Macau, China

**Keywords:** gamified HRM, gaming preference, intrinsic motivation, perceived organizational support, self-determination theory, work engagement

## Abstract

In the digital transformation era, maintaining employee work engagement has become a persistent challenge, as traditional incentive systems often fail to satisfy younger employees’ psychological needs for autonomy, competence, and relatedness. Gamified Human Resource Management (HRM) offers a novel means of enhancing work engagement through game-based mechanisms, yet prior studies have largely overlooked the mediating role of intrinsic motivation and the contextual contingencies shaping its effects. Drawing on self-determination theory (SDT), this study develops and empirically tests an integrated model using survey data collected from 418 respondents. The results of structural equation modeling reveal that gamified HRM significantly enhances work engagement, with intrinsic motivation partially mediating this relationship. Moreover, gaming preference and perceived organizational support moderate the positive effect of gamified HRM on engagement. These findings extend SDT by clarifying how gamified HR practices fulfill basic psychological needs and underscore that their effectiveness depends on the alignment between individual dispositions and organizational climate.

## 1 Introduction

With the continuous deepening of digital management paradigms, organizations are increasingly confronted with the pressing challenges of insufficient employee engagement and the diminishing effectiveness of traditional incentive mechanisms (Saraiva and Nogueiro, 2025). To address the heightened expectations of younger generations for enriched work experiences, a stronger sense of purpose, and greater autonomy, many firms have begun to incorporate gamification into human resource management practices, establishing new systems that emphasize motivational mechanisms, task challenges, and structured feedback loops (Silic et al., 2020). Importantly, gamified HRM does not simply trivialize work as entertainment; rather, it borrows the core logics of game design to stimulate positive emotions and foster sustained participation, thereby enhancing value co-creation between individuals and organizations (Cardador et al., 2017). Some scholars argue that games are uniquely capable of fulfilling deep psychological needs for control, competence, and social relatedness—needs often neglected in everyday organizational settings (Qian et al., 2022; Sailer et al., 2017). Others, however, warn that when entertainment logics infiltrate serious domains, they risk diluting meaning and critical reflection, reducing complex organizational issues to superficial or symbolic forms (Okwir and Nuur, 2022). Thus, an urgent question for contemporary management research is how organizations can leverage gamification to strengthen motivation while avoiding the potential trap of “over-entertainment.”

In recent years, research on gamified HRM has gained momentum, particularly in areas such as stimulating learning motivation, enhancing receptivity to performance feedback, and fostering knowledge sharing (Qian et al., 2022; Saraiva and Nogueiro, 2025). Self-determination theory (SDT) has become the dominant framework for explaining why gamification may be effective. SDT posits that the sustainability and quality of individual behavior depend fundamentally on the satisfaction of three basic psychological needs: autonomy, competence, and relatedness (Deci et al., 2017). When gamified design helps employees experience a sense of control, competence, and connectedness at work, they are more likely to shift from externally driven behavior to intrinsically motivated engagement, thereby achieving a stable and positive state of work involvement. Yet, the extant literature still faces clear limitations. Most studies emphasize the short-term effects of game elements, such as points or leaderboards, while neglecting whether employees’ engagement is genuinely driven by intrinsic motivation (Mekler et al., 2017; Silic et al., 2020). Moreover, individual acceptance of gamification varies substantially, but few studies explore how personal gaming preferences might moderate its effects. At the organizational level, the extent of institutional support and cultural inclusiveness may also determine whether gamification functions as a genuine motivational driver or remains a superficial administrative tool (Santos et al., 2021).

Addressing these gaps, this study develops an integrative model that combines SDT with contextual variables to examine how gamified HRM enhances employee work engagement through intrinsic motivation, while also considering the moderating roles of individual gaming preference and perceived organizational support. Specifically, it seeks to answer three key questions: (1) Does gamified HRM significantly improve employee work engagement? (2) Does intrinsic motivation mediate this relationship? (3) Do gaming preference and organizational support condition the strength of these effects? The study aims not only to clarify how gamification works but also to identify for whom and under what conditions it is most effective.

The theoretical contributions of this research are fourfold. First, it conceptualizes gamified HRM as an integrated institutional practice, moving beyond the fragmented focus on individual game elements and extending the theoretical boundaries of gamification in HRM. Second, by establishing intrinsic motivation as a mediating mechanism, it illuminates the psychological pathway linking institutional practices with employee behavioral outcomes, thereby enriching explanations of why gamification is effective and addressing the “black box” between management systems, motivation, and behavior. Third, by introducing gaming preference and perceived organizational support as moderators, this study highlights the dual importance of individual and organizational contexts, revealing the boundary conditions of gamification’s efficacy and expanding its situational dimension. Finally, by juxtaposing the optimistic view of gamification as a means of fulfilling deep psychological needs with critical concerns about the “entertainment trap,” the study provides empirical evidence that the two perspectives are not inherently contradictory but rather contingent upon the alignment of institutional design and organizational climate.

The remainder of this article is structured as follows: Section 1 introduces the background, research problems, objectives, and significance; section 2 reviews relevant literature on SDT, gamified HRM, and work engagement, and develops the research hypotheses; section 3 details the research design, sample selection, measurement, and analytical methods; section 4 presents the empirical results, including reliability and validity checks, hypothesis testing, and effect pathways; Section 5 discusses the theoretical and practical implications of the findings; and section 6 concludes with contributions, limitations, and directions for future research.

## 2 Literature review and hypotheses

### 2.1 The perspective of self-determination theory

Understanding how gamified human resource management (HRM) influences employee work engagement requires returning to the motivational foundation provided by self-determination theory (SDT) (Deci et al., 2017). SDT posits that the sustainability and quality of human behavior fundamentally depend on the satisfaction of three basic psychological needs: autonomy, competence, and relatedness (Neufeld, 2025; Vansteenkiste et al., 2020). When employees experience greater freedom of choice, perceive effectiveness in accomplishing tasks, and feel emotionally connected with others in the workplace, they are more likely to internalize external demands as voluntary actions, thereby demonstrating sustained engagement and positive motivation (Santos et al., 2021).

Over the past decades, SDT has been widely applied in organizational behavior and HRM research to explain how practices such as performance feedback, training systems, and leadership styles shape employee motivation (Olafsen et al., 2025). Within this framework, intrinsic motivation is not merely an outcome but serves as a critical mediating mechanism linking institutional practices with behavioral responses. In other words, the effectiveness of HRM initiatives hinges on their capacity to fulfill employees’ core psychological needs. If organizational practices remain confined to external incentives, behavioral effects tend to be short-lived; conversely, when day-to-day practices foster experiences of autonomy, competence, and relatedness, the resulting motivational outcomes are likely to be more enduring and profound (Qian et al., 2022).

### 2.2 Gamified HRM and its linkage with SDT

Gamification was initially conceived as a design approach in which point systems, levels, leaderboards, challenges, and rewards are introduced into non-game contexts to motivate individuals toward specific goals (Zhou et al., 2025). Within organizational management, gamified HRM is defined as the systematic integration of these elements into recruitment, training, performance appraisal, and employee development practices, with the aim of optimizing work experiences and stimulating motivation (Ortiz-Rojas et al., 2025). The essence of this practice does not lie in “making work entertaining,” but rather in leveraging interactive and feedback-oriented mechanisms to enhance psychological involvement, thereby transforming routine management activities into experiences that are more immersive and meaningful (Mekler et al., 2017).

From the perspective of self-determination theory (SDT), gamified HRM provides a practical pathway to satisfy employees’ three basic psychological needs. Goal setting and task challenges enable employees to gain a sense of mastery and achievement, thereby strengthening both autonomy and competence. Point and feedback systems offer immediate positive reinforcement, improving task clarity and perceived effectiveness, which further reinforces competence. Meanwhile, team-based competition and collaboration fulfill the need for relatedness, allowing employees to experience group identification and emotional support within the organization (Capatina et al., 2024). Empirical studies have shown that well-designed gamification mechanisms not only enhance learning motivation and receptivity to performance feedback but also foster deeper intrinsic motivation, which in turn translates into higher levels of work engagement and commitment (Ortiz-Rojas et al., 2025; Putranti et al., 2024).

Taken together, gamified HRM and SDT exhibit a natural alignment: the former, through concrete institutional and design practices, provides the practical vehicle for satisfying psychological needs; the latter, in turn, offers the theoretical foundation for explaining why gamification can effectively drive employee behavior. This integration not only illustrates *how* gamification operates but also underscores *why* psychological mechanisms are indispensable for its success.

### 2.3 Gamified HRM and work engagement

Work engagement is commonly defined as a positive and enduring psychological state characterized by vigor, dedication, and absorption (González-Romá and Bakker, 2002). Engaged employees are not only emotionally and cognitively connected to their work but also display sustained energy and high levels of task concentration in their behavior. A growing body of research indicates that work engagement is positively associated with individual wellbeing and organizational commitment, while also serving as a critical driver of organizational performance, innovation, and long-term competitive advantage (Rabuana and Yanuar, 2023).

Against this backdrop, gamified HRM has been regarded as an effective pathway to enhance employee engagement. First, goal orientation and task challenges provide employees with a clear direction and measurable sense of accomplishment, thereby stimulating persistent motivation. Second, point systems, feedback, and reward mechanisms acknowledge employees’ efforts in a timely manner, strengthening their sense of competence and enabling them to sustain high levels of energy and focus. Third, team-based collaboration and competitive elements commonly embedded in gamification foster interaction and cohesion, thereby fulfilling employees’ needs for belonging and recognition (Capatina et al., 2024; Ortiz-Rojas et al., 2025; Putranti et al., 2024; Zhou et al., 2025). In short, gamified HRM systematically satisfies the needs for autonomy, competence, and relatedness, offering employees a comprehensive psychological support structure that underpins deeper and more sustained engagement.

Preliminary empirical evidence lends further support to this logic. Studies have shown that embedding gamified elements into HRM systems can significantly enhance employee satisfaction and commitment (Mekler et al., 2017), while experimental findings suggest that specific gamification design features directly boost engagement by fulfilling psychological needs (Capatina et al., 2024; Sailer et al., 2017). Based on these insights, it can be hypothesized that when organizations adopt gamified HRM to create more immersive and meaningful work experiences, employees will demonstrate higher levels of engagement.

*H1*: Gamified HRM has a positive effect on employee work engagement.

### 2.4 The mediating role of intrinsic motivation

Although gamified HRM can directly enhance employees’ work engagement, a more critical mechanism lies in whether it can successfully be transformed into intrinsic motivation. Intrinsic motivation refers to the psychological drive that prompts individuals to engage in activities spontaneously out of interest, curiosity, or value identification (Di Domenico and Ryan, 2017). Unlike short-term behaviors driven by external rewards, actions fueled by intrinsic motivation are generally more enduring and of higher quality, as individuals derive satisfaction and enjoyment from the activity itself (Van den Broeck et al., 2021).

Within the SDT framework, intrinsic motivation is regarded as the direct outcome of satisfying the three basic psychological needs. When employees experience greater autonomy, a stronger sense of competence, and enhanced relatedness with colleagues through gamified HRM practices, they are more likely to internalize external requirements as voluntary choices. For instance, challenges and feedback mechanisms not only make employees more aware of their progress but also encourage them to engage in tasks because they want *to* rather than because they have to Grenier et al. (2024). This transformation of motivation is the key to sustained engagement, as it indicates that employees’ efforts are based on self-determination rather than external pressure.

Empirical research has provided support for this logic. Some studies show that the effectiveness of gamified management systems largely depends on whether they can stimulate employees’ intrinsic interest and sense of value (Liu and Gao, 2025). Others demonstrate that gamification mechanisms possess unique advantages in facilitating the internalization of motivation, thereby explaining why employees exposed to similar institutional arrangements may exhibit varying degrees of engagement (Magioli Sereno and Ang, 2024). Hence, intrinsic motivation should not be regarded as a peripheral variable, but rather as a central psychological channel through which gamified HRM influences work engagement.

*H2*: Intrinsic motivation mediates the relationship between gamified HRM and employee work engagement.

### 2.5 The moderating roles of individual differences and organizational support

Although gamified HRM is theoretically capable of enhancing employee engagement, its effects are not uniform across all individuals. Personal differences may influence how employees perceive and accept gamification mechanisms. One particularly salient factor is individual gaming preference. Prior studies suggest that employees exhibit differentiated preferences when confronted with competitive, cooperative, exploratory, or achievement-oriented game elements (Capatina et al., 2024). For example, employees with a preference for competition may experience stronger feelings of competence and control when exposed to leaderboards or rivalry mechanisms, whereas those with a preference for cooperation may derive a stronger sense of relatedness when engaging in team-based tasks and collaborative settings. When gamified HRM aligns with such personal preferences, employees’ psychological needs are more easily satisfied and converted into intrinsic motivation. Conversely, when preferences and gamification designs are mismatched, employees may perceive gamification as external pressure, thereby weakening their sense of engagement. Hence, individual gaming preference is likely to serve as a critical boundary condition shaping the effectiveness of gamified HRM (Bitrián et al., 2023).

*H3*: Individual gaming preference moderates the relationship between gamified HRM and employee work engagement, such that the relationship is stronger when preference levels are high.

At the organizational level, perceived organizational support (POS) also plays a decisive role in determining the actual effectiveness of gamified HRM. POS refers to the extent to which employees perceive that the organization values their contributions and cares about their wellbeing (Eisenberger et al., 1986; Li et al., 2022). When organizational systems, cultural atmosphere, and resource allocation send strong signals of support, employees are more inclined to regard gamification mechanisms as positive tools for growth and development rather than mere instruments of control. In contrast, in environments lacking sufficient organizational support, gamification—even if present—may be dismissed as a superficial or symbolic management tool, unlikely to generate genuine intrinsic motivation. Previous research has demonstrated that organizational support significantly enhances employees’ acceptance of institutional initiatives and facilitates motivational internalization (Grenier et al., 2024; Habib et al., 2025). This implies that POS may serve as an important moderator in the pathway linking gamified HRM to employee engagement.

*H4*: Perceived organizational support moderates the relationship between gamified HRM and employee work engagement, such that the relationship is stronger when perceived organizational support is high.

Taken together, this study builds on SDT to explore how gamified HRM enhances employee engagement by stimulating intrinsic motivation, while further considering the boundary conditions at both individual and organizational levels. The four hypotheses collectively form an integrated research model: gamified HRM is conceptualized as the independent variable, employee engagement as the dependent variable, intrinsic motivation as the mediator, and gaming preference and perceived organizational support as moderators.

This model highlights a multi-level logic of influence: on one hand, gamified HRM enhances intrinsic motivation by satisfying employees’ needs for autonomy, competence, and relatedness, thereby promoting engagement; on the other hand, the effectiveness of this pathway is not homogeneous but is jointly shaped by individual differences in preferences and the organizational climate. In other words, the extent to which gamified HRM translates into high levels of work engagement depends on whether employees “like this approach” and whether organizations “support this approach.”

**FIGURE 1:**
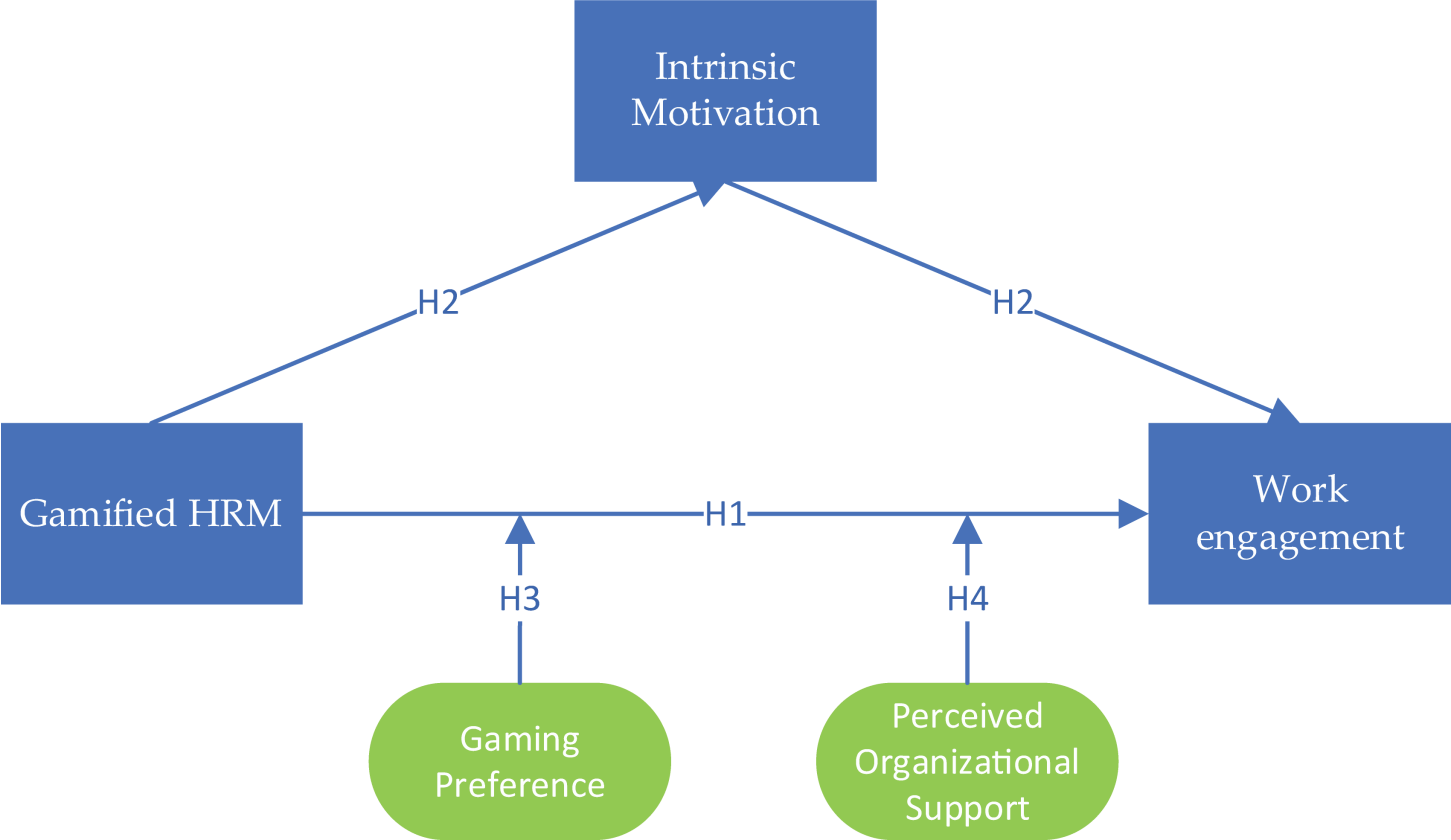
Hypothesis framework.

Based on this logic, the Hypothesis framework of this study is presented in Figure 1.

## 3 Methodology

### 3.1 Research design

This study adopted a cross-sectional survey design to test the proposed hypotheses. Such an approach has been widely applied in organizational behavior and human resource management research, as it enables the collection of large-scale frontline data from multiple firms within a relatively short period of time, thereby ensuring that the findings possess both representativeness and generalizability (Setia, 2016). Considering that the application of gamified HRM may vary substantially across organizations, the cross-sectional design allowed us to capture general patterns across different industries and organizational contexts, while also providing the necessary data foundation for subsequent statistical modeling and hypothesis testing.

To ensure measurement rigor and reduce common method bias (CMB), several procedural remedies were incorporated into the research design. First, some survey items were reverse-coded to prevent mechanical responding. Second, varying response anchors were used across different sections of the questionnaire to minimize systematic bias arising from habitual answering. Third, respondents were explicitly assured of anonymity and confidentiality during survey administration, thus alleviating concerns about managerial traceability and mitigating evaluation apprehension or social desirability effects (Craighead et al., 2011).

To ensure consistency in measurement and maintain analytical rigor, all data were collected through a single standardized questionnaire administered to individual respondents. This approach allowed the study to capture employees’ perceptions of gamified HRM practices, intrinsic motivation, work engagement, and related contextual variables within a unified measurement framework, thereby avoiding discrepancies that may arise from heterogeneous data sources. All data were collected during the same survey period to ensure procedural consistency. Respondents completed the questionnaire voluntarily and independently, and no temporal separation was required because the study relied on a single-source, single-time-point design consistent with many prior works in HRM and organizational behavior.

### 3.2 Sample and data collection

To ensure both the representativeness of the sample and the authenticity of the data, this study collected information from firms and employees through three channels: (1) enterprises collaborating with the research team, particularly those that had already introduced digital or gamified mechanisms into their HRM practices; (2) industry associations and professional training institutions, which facilitated questionnaire distribution to their member firms; and (3) executive education and continuing education programs at selected universities, whose participants—mainly corporate managers or middle-level executives—enabled access to employees within their organizations.

During implementation, a single standardized questionnaire was distributed to individual respondents across participating organizations. This instrument captured perceptions of gamified HRM practices, intrinsic motivation, work engagement, gaming preference, and perceived organizational support. Collecting all data from a unified survey ensured consistency in measurement and avoided discrepancies that may arise from heterogeneous data sources or different respondent roles (Zhang et al., 2024).

Rigorous procedures were applied to ensure data quality. Questionnaires with excessive missing values or uniformly patterned responses were removed, and all cases were screened for completeness, consistency, and outliers. After data cleaning, a total of 418 valid questionnaires were retained. Among the respondents, 54.8% were male and 45.2% were female. The average age was 34.39 years (SD = 6.31), and the average organizational tenure was 10.96 years (SD = 7.11). Regarding educational background, 12.2% held a master’s degree or above, 41.63% held a bachelor’s degree, 32.06% had a college diploma, and 14.11% had a high school education or below. This distribution reflects the demographic structure of a broad cross-section of the contemporary workforce in China, including both knowledge-intensive roles and operational positions in manufacturing and service sectors. The diversity of the sample enhances the representativeness and generalizability of the study’s findings (Podsakoff et al., 2003).

### 3.3 Measurement of variables

All core constructs in this study were measured using scales that have been widely applied and validated in leading international journals, with a translation–back translation procedure employed to ensure cultural and linguistic equivalence. All items were assessed on a five-point Likert scale (1 = strongly disagree, 5 = strongly agree).

#### 3.3.1 Gamified HRM

A seven-item scale developed by Ērgle and Ludviga was used to assess the extent to which gamification mechanisms were implemented in recruitment, training, performance appraisal, and communication practices (Ērgle and Ludviga, 2018). A sample item is: “The company uses gamified mechanisms in employee training programs.”

#### 3.3.2 Work engagement

Work engagement was measured using the Utrecht Work Engagement Scale (UWES), which captures three dimensions: vigor, dedication, and absorption (Schaufeli et al., 2002). A sample item is: “I am fully absorbed in the task I am currently engaged in.”

#### 3.3.3 Intrinsic motivation

Intrinsic motivation was measured with the Intrinsic Motivation Inventory (IMI) (Ryan, 1982). A sample item is: “I find doing this activity interesting in itself.”

#### 3.3.4 Gaming preference

Gaming preference refers to employees’ orientation toward specific gamified design features embedded in human resource management practices. Employees’ preferences for competitive, cooperative, and achievement-oriented game mechanisms were measured with self-developed items based on prior research (Hauge et al., 2023). A sample item is: “I enjoy motivating myself through competition.”

#### 3.3.5 Perceived organizational support

Perceived organizational support was assessed using the scale developed by Eisenberger and colleagues (Eisenberger et al., 1986). A sample item is: “The organization cares about my needs.”

#### 3.3.6 Control variables

Following prior studies, demographic factors such as gender, age, education level, and organizational tenure were included as control variables (Bernerth and Aguinis, 2016).

A pilot test indicated that all scales demonstrated satisfactory reliability, with Cronbach’s α coefficients above 0.80, suggesting a high level of internal consistency (Taber, 2018).

### 3.4 Data analysis methods

Data analysis was conducted in three stages. First, descriptive statistics and correlation analyses were performed using SPSS 26.0 to provide preliminary insights into the relationships among the variables. Second, confirmatory factor analysis (CFA) was conducted with AMOS 24.0 to examine the convergent and discriminant validity of the measurement scales. Model fit was evaluated using standard indices such as χ^2^/df, CFI, TLI, and RMSEA. Finally, the hypothesized relationships were tested through structural equation modeling (SEM), allowing for a comprehensive examination of the proposed research model.

The mediation effects were assessed using the bootstrapping method with 5,000 resamples, and significance was determined based on 95% bias-corrected confidence intervals. Moderation effects were examined using hierarchical regression analyses with interaction terms, supplemented by simple slope analyses to illustrate the direction and strength of the interactions. A significance level of 0.05 was adopted for all statistical tests.

## 4 Data analysis and results

### 4.1 Data preprocessing

Before proceeding to hypothesis testing, the dataset was systematically preprocessed to ensure the reliability and rigor of subsequent analyses. First, missing values were examined, and the proportion of missing data for all variables was found to be below 5%, which falls within the commonly accepted threshold in academic research (Madley-Dowd et al., 2019). Accordingly, mean imputation was applied to address missing data and maximize the sample size. Second, potential outliers were identified using standardized residuals and boxplot diagnostics, resulting in the removal of four extreme cases. After data cleaning, a total of 418 valid questionnaires were retained, yielding an effective response rate of 81.3% and ensuring high overall data quality. To assess common method bias (CMB), Harman’s single-factor test was conducted. The exploratory factor analysis showed that the largest single factor accounted for 32.6% of the variance, remaining well below the recommended 40% threshold, suggesting that CMB is unlikely to substantially bias the results. In addition to Harman’s single-factor test, a single-factor CFA model was estimated to further assess common method bias. The single-factor model showed inadequate fit indices (χ^2^/df > 5, CFI < 0.80, RMSEA > 0.10), indicating that a single latent factor could not adequately explain the covariance among the measured constructs. This result also suggests that common method bias is unlikely to substantially contaminate the observed relationships. Meanwhile, several procedural remedies—including anonymous participation, reverse-coded items, and varied response formats—had been incorporated into the survey design to further mitigate the risk of common-source bias. Finally, the demographic characteristics of the sample were summarized (see Table 1). The distribution of gender, age, educational background, and tenure was balanced and broadly consistent with the workforce composition in many domestic industries, supporting the representativeness of the sample.

**TABLE 1:** Sample characteristics (*N* = 418).

Variable	Value
Gender (male)	54.8%
Gender (female)	45.2%
Average age	34.39 years (SD = 6.31)
Average tenure	10.96 years (SD = 7.11)
Education—master and above	12.2%
Education—bachelor	41.63%
Education—college	32.06%
Education—high school	14.11%

### 4.2 Descriptive statistics and correlation analysis

Before conducting reliability and validity assessments and hypothesis testing, descriptive statistics and correlation analyses were performed on the main variables. Means (M), standard deviations (SD), and Pearson correlation coefficients were calculated to provide an initial understanding of the strength and direction of the relationships among variable.

The results are presented in Table 2. Overall, gamified HRM, intrinsic motivation, perceived organizational support, gaming preference, and work engagement were all positively correlated. This finding aligns with the logic of self-determination theory (SDT), which suggests that when organizations satisfy employees’ needs for autonomy, competence, and relatedness through gamification mechanisms, intrinsic motivation is more likely to be activated, thereby fostering higher levels of work engagement.

**TABLE 2:** Descriptive statistics and correlations.

Variable	M	SD	1	2	3	4	5
1. Gamified HRM	3.12	0.78	1				
2. Intrinsic motivation	3.85	0.66	0.42*	1			
3. Work engagement	3.97	0.71	0.38*	0.51*	1		
4. Gaming preference	3.45	0.82	0.29	0.33***	0.31**	1	
5. Perceived org. support	3.68	0.74	0.36*	0.40*	0.39*	.27	1

*N* = 418, *, **, and *** indicate significant at the 10, 5, and 1% levels, respectively.

Moreover, control variables such as gender, age, education, and tenure were not significantly correlated with the core constructs, indicating that demographic characteristics did not substantially bias the results.

From the correlation analysis, several important patterns emerge:

1. Gamified HRM is positively correlated with work engagement (*r* = 0.38, *p* < 0.001), providing preliminary support for Hypothesis 1.
2. Gamified HRM is positively correlated with intrinsic motivation (*r* = 0.42, *p* < 0.001), laying the empirical foundation for its mediating role.
3. Intrinsic motivation is positively correlated with work engagement (*r* = 0.51, *p* < 0.001), consistent with theoretical expectations.
4. Both individual differences (gaming preference) and organizational context (perceived organizational support) are positively correlated with the core constructs, suggesting their potential as moderators.

Taken together, the descriptive statistics and correlation results not only confirm the basic logic of the proposed relationships but also provide empirical support for subsequent reliability testing and structural equation modeling analyses.

### 4.3 Reliability and validity analysis

Before proceeding with hypothesis testing, the reliability and validity of the measurement instruments were examined to ensure both consistency and structural soundness. Reliability analysis assessed the internal consistency of the scales, while validity analysis focused on convergent and discriminant validity to ensure that each latent construct was accurately captured by its indicators and sufficiently distinct from other constructs.

#### 4.3.1 Reliability analysis

Cronbach’s α coefficients and composite reliability (CR) were used as indicators of internal consistency. A Cronbach’s α value above 0.70 is generally considered acceptable, while a CR above 0.70 indicates stability in measurement results. As shown in Table 3, all variables in this study had Cronbach’s α values above 0.87, with the highest reaching 0.93. Similarly, CR values were all above 0.88, significantly exceeding the recommended threshold. These results suggest that the scales demonstrated excellent internal consistency and reliably reflected the intended constructs.

**TABLE 3:** Reliability and convergent validity results.

Construct	Items	Cronbach’s α	CR	AVE
Gamified HRM	7	0.91	0.92	0.63
Intrinsic motivation	6	0.89	0.90	0.61
Work engagement	6	0.93	0.94	0.66
Gaming preference	5	0.87	0.88	0.59
Perceived organizational support	8	0.90	0.91	0.62

#### 4.3.2 Convergent validity

Convergent validity was assessed using confirmatory factor analysis (CFA). Standardized factor loadings (SFLs) exceeded 0.60 and were statistically significant, while the average variance extracted (AVE) values for all constructs were greater than 0.50, indicating that the majority of variance in the latent constructs was explained by their observed indicators. As reported in Table 3, factor loadings ranged from 0.65 to 0.87, and AVE values were all above 0.59, satisfying the criteria for convergent validity.

#### 4.3.3 Discriminant validity

Discriminant validity was further assessed using the Fornell–Larcker criterion, which requires that the square root of each construct’s AVE exceed its correlations with other constructs. Table 4 presents the results: the diagonal values (√AVE) were consistently higher than the off-diagonal correlations. For example, the square root of AVE for work engagement (0.81) was greater than its correlations with gamified HRM (*r* = 0.38) and intrinsic motivation (*r* = 0.51), confirming discriminant validity.

**TABLE 4:** Discriminant validity results (Fornell–Larcker criterion).

Variable	1	2	3	4	5
1. Gamified HRM	**0.79**				
2. Intrinsic motivation	0.42	**0.78**			
3. Work engagement	0.38	0.51	**0.81**		
4. Gaming preference	0.29	0.33	0.31	**0.77**	
5. Perceived org. support	0.36	0.40	0.39	0.27	**0.79**

Diagonal values (in bold) represent the square roots of the average variance extracted (AVE).

Taken together, the results confirm that the measurement scales used in this study met international standards of reliability (Cronbach’s α and CR), convergent validity (factor loadings and AVE), and discriminant validity (Fornell–Larcker criterion). This provides a solid measurement foundation for subsequent SEM analyses and hypothesis testing.

### 4.4 Hypothesis testing

#### 4.4.1 Main effect and mediation effect test

To test Hypothesis 1, gamified HRM was specified as the independent variable and work engagement as the dependent variable in the structural equation model (SEM). The results showed that gamified HRM exerted a significant positive effect on employee work engagement (β = 0.31, *p* < 0.001). This finding provides initial support for the theoretical assumption that when organizations integrate points, challenges, and feedback mechanisms into recruitment, training, and performance evaluation processes, employees’ vigor, dedication, and absorption are significantly enhanced.

To examine Hypothesis 2, which posited that intrinsic motivation mediates the relationship between gamified HRM and work engagement, a bootstrapping approach with 5,000 resamples was employed. The results revealed a significant indirect effect [β = 0.18, 95% CI = (0.09, 0.29)], with the confidence interval excluding zero. This indicates that intrinsic motivation partially mediated the relationship. In other words, gamified HRM enhances employees’ sense of autonomy, competence, and relatedness, thereby strengthening intrinsic motivation and ultimately promoting higher levels of engagement. The relevant path coefficients are presented in Table 5.

**TABLE 5:** Results of hypothesis testing (SEM and bootstrapping).

Path	β	SE	95% CI (lower, upper)	Supported
H1: Gamified HRM → Work Engagement	0.31*	0.07	(0.17, 0.45)	Yes
H2: Gamified HRM → intrinsic motivation → work engagement	0.18*	0.05	(0.09, 0.29)	Yes
H3: Gamified HRM × gaming preference → work engagement	0.15**	0.06	(0.05, 0.26)	Yes
H4: Gamified HRM × POS → work engagement	0.17***	0.07	(0.04, 0.31)	Yes

*, **, and *** indicate significant at the 10, 5, and 1% levels, respectively.

#### 4.4.2 Moderation effect test

The moderating roles of individual and organizational contexts were further examined.

##### 4.4.2.1 Gaming preference

**FIGURE 2:**
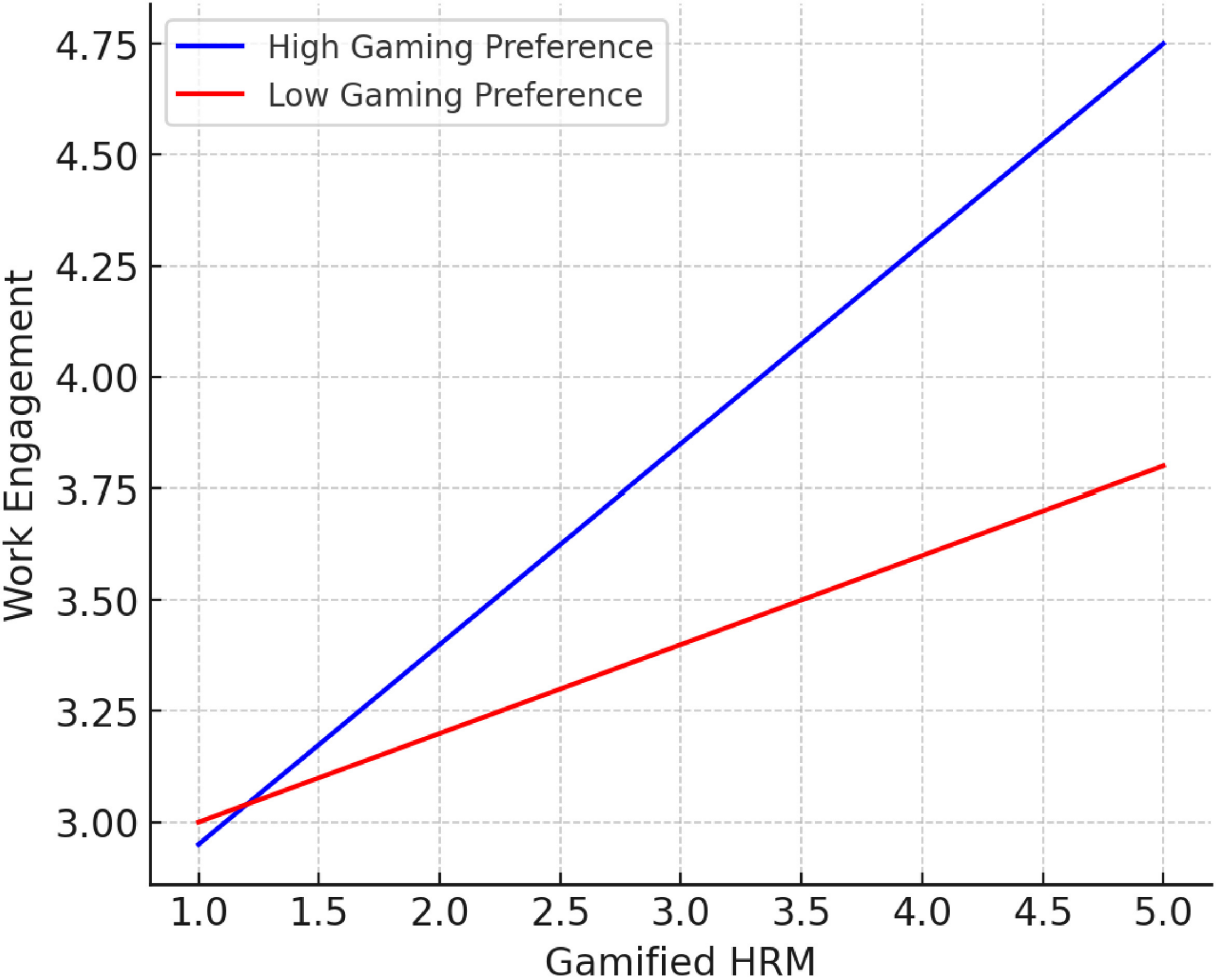
Moderating effect of gaming preference.

The interaction analysis showed that the interaction between gamified HRM and gaming preference significantly predicted work engagement (β = 0.15, *p* < 0.01). Simple slope tests indicated that the positive effect of gamified HRM on work engagement was stronger among employees with high gaming preference, whereas the effect was weaker among those with low preference (see Figure 2 for details). Thus, Hypothesis 3 was supported.

##### 4.4.2.2 Perceived organizational support

**FIGURE 3:**
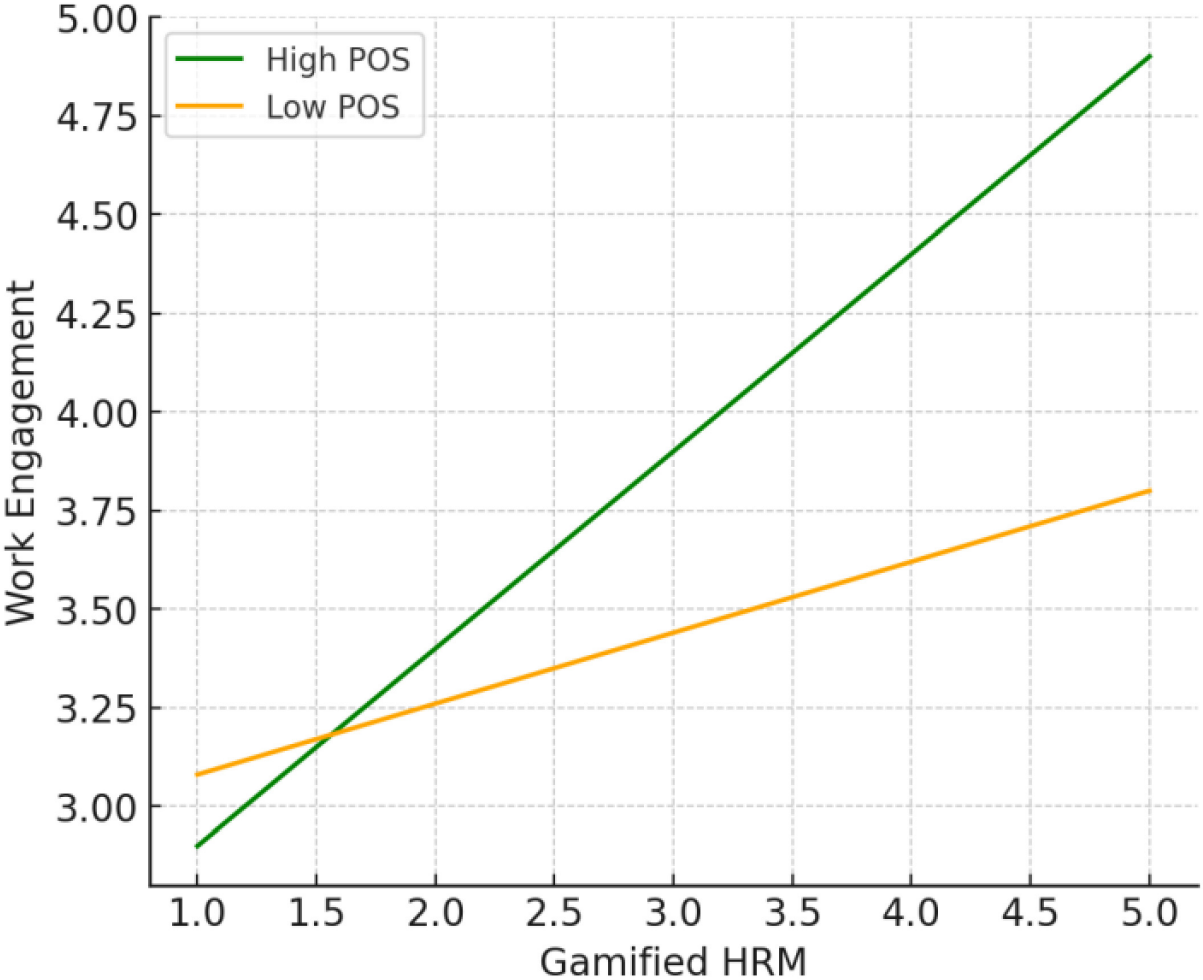
Moderating effect of perceived organizational support.

The interaction analysis also revealed that the interaction between gamified HRM and POS was significant (β = 0.17, *p* < 0.01). Further analysis showed that when POS was high, gamified HRM was more likely to be interpreted by employees as a tool for growth and development, significantly enhancing engagement. In contrast, when POS was low, the effectiveness of gamification mechanisms was notably weaker (refer to Figure 3). Thus, Hypothesis 4 was supported.

In summary, Hypotheses H1–H4 were all empirically supported. Gamified HRM not only directly enhanced employee engagement but also indirectly promoted engagement through intrinsic motivation. Moreover, the strength of these effects was significantly conditioned by individual-level gaming preference and organizational-level support. These findings provide robust evidence for the proposed theoretical model and clarify the boundary conditions of gamification in HRM across different contexts.

### 4.5 Robustness check

To ensure the reliability and generalizability of the findings, several robustness checks were conducted from the perspectives of alternative dependent variables, subgroup analysis, and potential method bias. First, at the dependent variable level, the three sub-dimensions of work engagement—vigor, dedication, and absorption—were separately included in regression models. The results indicated that gamified HRM exerted significant positive effects across all three dimensions, with the mediating role of intrinsic motivation remaining significant. This suggests that the conclusions are not contingent upon a single measurement indicator. Second, subgroup analyses were performed by dividing the sample according to age and educational level. The results showed consistent effect directions across groups, and the core pathway (Gamified HRM → Intrinsic Motivation → Work Engagement) remained significant, demonstrating the stability of the model across demographic characteristics. Finally, diagnostic tests confirmed that all variance inflation factor (VIF) values were below 2, suggesting that multicollinearity was not a serious concern. In addition, both the single-factor CFA model and Harman’s single-factor test results indicated that common method bias (CMB) was not a substantial threat.

The summary of robustness checks is presented in Table 6. As shown, under various alternative specifications and methodological controls, the key conclusions remained consistent, thereby further reinforcing the credibility and external validity of this study.

**TABLE 6:** Results of robustness checks.

Test method	Key findings	Conclusion
Alternative dependent variables (vigor, dedication, absorption)	Gamified HRM shows significant positive effects across all dimensions; mediation by intrinsic motivation remains	Results remain robust
Subgroup analysis (age, education level)	Effect directions consistent; core path remains significant and stable	Results remain robust
Multicollinearity test (VIF < 2)	No serious multicollinearity issues identified	Results remain robust
Harman’s single-factor test and CFA	Poor fit for single-factor model; CMB not a major concern	Results remain robust

## 5 Discussion

### 5.1 Discussion of main findings

This study found that gamified HRM has a significant positive effect on employee work engagement, a result largely consistent with prior research. For instance, gamification mechanisms have been shown to enhance employee satisfaction and commitment through immediate feedback and task-based incentives (Capatina et al., 2024; Putranti et al., 2024). Experimental studies have further demonstrated that design elements such as points and leaderboards directly satisfy employees’ needs for autonomy, competence, and relatedness, thereby fostering higher levels of engagement (Li et al., 2024). By obtaining similar findings in a different cultural context, this study confirms the cross-contextual applicability of gamified HRM. However, the effect size in this study (β = 0.31) is somewhat smaller than that reported in some Western studies (often above 0.40). A plausible explanation is that gamified practices in many Chinese firms remain in their exploratory stages, and employees may not yet fully recognize or embrace such innovative systems, thereby weakening the overall motivational impact.

With respect to the mediating role of intrinsic motivation, the findings of this study align closely with the argument that the effectiveness of gamification hinges on its ability to facilitate the internalization of extrinsic incentives into intrinsic motivation (Di Domenico and Ryan, 2017; Li et al., 2024). Unlike some prior research, however, this study observed a partial—rather than full—mediation effect. This suggests that gamified HRM can influence engagement not only by enhancing intrinsic motivation but also through a direct pathway. Such divergence may stem from methodological and sample differences: this study relied on cross-sectional matched data, whereas prior experimental studies often employed more refined designs and stronger manipulations, which may more easily activate intrinsic motivation as the sole mechanism. Future research may therefore benefit from longitudinal or experimental designs to further validate the causal pathways of gamified practices across organizational contexts.

At the individual level, this study identified a significant moderating effect of gaming preference, echoing earlier findings that motivational responses to gamified features vary by individual differences in gaming orientation (Hauge et al., 2023; Mekler et al., 2017; Xiao and Hew, 2024). Unlike previous work, however, our results revealed that employees with low gaming preference were not entirely unaffected; instead, they still experienced modest gains in engagement. This indicates that even when employees are not naturally inclined toward gaming elements, institutionalized designs may still guide behavior to some extent, albeit with weaker effects. Such findings extend prior conclusions and suggest that organizations should tailor gamified HRM to heterogeneous employee preferences in order to maximize its overall effectiveness.

At the organizational level, the results underscore the critical role of perceived organizational support (POS) as a boundary condition. Consistent with classic perspectives emphasizing that organizational support enhances trust and perceived value of institutional initiatives (Eisenberger et al., 1986; Peng et al., 2023), this study further demonstrates that in contexts lacking sufficient support, gamified HRM not only loses effectiveness but may also be perceived by employees as a superficial or symbolic tool. This observation resonates with concerns about the “entertainment trap,” wherein entertainment logics dilute meaning and critical engagement in serious domains (Li et al., 2022). The implication here is that the success of gamified HRM depends not solely on its design features but also on its alignment with broader organizational climate and support structures.

### 5.2 Extended findings

Beyond the testing of core hypotheses, this study revealed several additional phenomena that merit further discussion.

First, a comparison of effect pathways suggests that gamified HRM not only exerted a direct influence on employee engagement but also operated indirectly through intrinsic motivation. In other words, when employees were exposed to mechanisms such as points, feedback, and challenges, they exhibited higher levels of engagement both because of the novelty and interactivity of the system itself, and because, when these mechanisms addressed their needs for autonomy, competence, and relatedness, they gradually transformed into more stable intrinsic motivation that sustained long-term engagement. This dual pathway of “immediate stimulation” and “deeper transformation” has rarely been tested simultaneously in prior studies, which have tended to emphasize the process of motivational internalization alone (Habib et al., 2025; Wibisono et al., 2023). The findings here suggest that short-term direct effects of gamification should not be overlooked. One plausible reason is that many of the firms in this study were in the early stages of implementing gamified mechanisms, where the novelty of design features may itself be sufficiently motivating.

Second, the moderating roles of individual differences and organizational context displayed notable distinctions. Gaming preference primarily functioned as a “magnifier effect”: employees with strong preferences benefited more, while those with weaker preferences still experienced modest improvements. By contrast, perceived organizational support (POS) operated as a form of “collective enhancement”: when employees broadly perceived organizational care and support, the positive effects of gamification were amplified across the entire group. This divergence indicates that individual-level and organizational-level factors are complementary in shaping the outcomes of gamified HRM—the former emphasizing person-specific fit, the latter emphasizing contextual alignment. This insight suggests that future studies should consider both perspectives simultaneously when evaluating the utility of gamified practices (Liu and Gao, 2025; Qiao et al., 2025).

Third, robustness checks revealed that the effects of gamified HRM on the three dimensions of work engagement were not entirely uniform. Its impact on vigor was the strongest, whereas its effects on dedication and absorption were positive but relatively weaker. This pattern is consistent with the conceptual nature of engagement: vigor reflects immediate energy and enthusiasm, which are more easily influenced by novel systems and situational stimuli; dedication and absorption, in contrast, often require deeper value alignment and long-term cultural reinforcement that cannot be achieved solely through mechanisms in the short run (Wibisono et al., 2023). These findings suggest that gamified HRM may be more suitable for stimulating short-term participation and energy, while long-term engagement necessitates integration with organizational culture and value systems.

Finally, the results provide new evidence in response to concerns about the so-called “entertainment trap.” While Postman (1985) warned that the infiltration of entertainment logics into serious domains may dilute meaning and critical spirit (Okwir and Nuur, 2022), our findings suggest otherwise: when gamified designs align with employees’ psychological needs and are embedded in a supportive organizational climate, they can foster higher-quality engagement rather than superficial compliance. This indicates that entertainment logics per se are not inherently problematic; the critical factor lies in whether organizations can balance playfulness with meaningfulness in their institutional designs.

Overall, these extended findings enrich our understanding of how gamified HRM operates: it entails both direct motivational effects and deeper transformations through intrinsic motivation; its boundary conditions manifest differently at the individual and organizational levels; and its influence varies across dimensions of engagement. These patterns not only supplement gaps in the existing literature but also provide specific entry points for future research.

## 6 Conclusion

### 6.1 Summary of findings

Drawing on self-determination theory (SDT), this study investigated how gamified human resource management (HRM) influences employee work engagement through intrinsic motivation, while further examining the moderating roles of individual gaming preference and perceived organizational support. Based on survey data from 418 respondents, structural equation modeling and multiple robustness checks were conducted. The results yield several key conclusions: (1) gamified HRM significantly enhances employee work engagement; (2) intrinsic motivation serves as a partial mediator between gamified HRM and work engagement; (3) both individual-level differences (gaming preference) and organizational-level factors (perceived organizational support) positively moderate the core pathway, though their modes of influence differ; and (4) the findings remain robust across alternative measurements and subsamples, thereby reinforcing the reliability and generalizability of the results.

### 6.2 Theoretical contributions

This study advances the theoretical understanding of gamified HRM in several ways. By approaching gamification as a holistic institutional practice rather than focusing on individual elements, it situates gamified HRM more firmly within the literature on organizational incentive mechanisms. Earlier work tended to isolate features such as points or leaderboards, while overlooking the systemic power that emerges when these features are integrated into a coherent HRM framework.

The findings also clarify the role of intrinsic motivation. Existing research has often emphasized the importance of motivational internalization, yet the evidence here shows that the mechanism is only partial. Gamified practices can foster engagement not only through the fulfillment of psychological needs but also by directly stimulating behavioral responses. This offers more fine-grained evidence for understanding how institutional practices are linked to motivation and behavior.

Attention is further drawn to the conditions under which gamified HRM is most effective. The moderating effects of gaming preference and perceived organizational support demonstrate that individual and organizational contexts shape outcomes in distinct ways. Preference reflects the degree of alignment between personal interests and system design, while support reflects whether the organizational climate provides legitimacy and reinforcement for gamification. Such findings extend the literature on contextual contingencies and point to the importance of cross-level perspectives.

The results also contribute to an ongoing academic debate. Scholars such as McGonigal argue that games can awaken fundamental psychological needs, whereas Postman warns that entertainment logics may erode meaning and seriousness in institutional life. Evidence from this study suggests that the two positions are not inherently contradictory. Gamification may generate meaningful engagement when institutional design is aligned with both organizational support and employees’ psychological needs, but risks becoming superficial when such alignment is absent.

### 6.3 Practical implications

The findings offer several implications for managerial practice. For firms, the introduction of gamified HRM can serve as an effective tool for attracting and energizing younger employees. When applied to recruitment, training, or performance feedback, gamification has the potential to enhance interaction and participation. Its value, however, does not lie in surface-level excitement, but in the extent to which the mechanisms genuinely address employees’ psychological needs.

From the perspective of managers, gamified mechanisms should not be designed as mere external incentives. The emphasis needs to be placed on creating experiences that allow employees to feel autonomy, competence, and relatedness. Only when these needs are met can external arrangements be transformed into sustainable intrinsic motivation.

The results also highlight the heterogeneity of employee responses to gamification. Not all employees embrace such mechanisms equally, which suggests that firms should provide diverse and flexible forms of gamification to accommodate different preferences. Organizational climate plays a decisive role as well: without a supportive environment, even sophisticated designs may be dismissed as superficial or manipulative, undermining their motivational potential.

Another insight is that gamified HRM is particularly effective in stimulating short-term energy and engagement but less sufficient for cultivating long-term and stable commitment. For durable effects, gamification must be integrated with broader organizational practices, including cultural development, value alignment, and career advancement opportunities. In this sense, gamification can function as a powerful entry point, but it is not a substitute for deeper and more enduring forms of management.

### 6.4 Limitations and future research directions

Despite its theoretical and practical contributions, this study has several limitations that warrant attention. The data were collected through a cross-sectional survey. Although both procedural and statistical remedies were applied, common method bias cannot be entirely ruled out due to the cross-sectional and single-source nature of the data. Future studies may employ longitudinal or multi-source research designs to further strengthen causal inference. The sample was drawn primarily from firms in eastern China. Cultural and institutional environments may have influenced the findings, raising questions about generalizability. Comparative studies across different cultural contexts would provide a stronger basis for assessing the robustness of the conclusions. The analysis focused on the moderating roles of individual gaming preference and perceived organizational support. Other contextual variables—such as leadership styles, organizational climate, or the degree of digitalization—could be introduced in future studies to uncover additional boundary conditions. Finally, this study concentrated on work engagement as the primary outcome variable. Yet gamified HRM may also shape a broader set of employee outcomes, including innovative behavior, knowledge sharing, and even wellbeing. Taken together, these findings deepen our understanding of how gamified HRM shapes employee motivation and engagement, and they point toward a broader research agenda for unpacking the motivational dynamics of digital HR practices across different organizational and cultural contexts.

## Data Availability

The original contributions presented in this study are included in this article/Supplementary material, further inquiries can be directed to the corresponding author.
