# Supplementary material for: Gamified human resource management as a driver of employee engagement through intrinsic motivation
Source: Front Psychol. 2026 Jan 21;16:1746973. doi: 10.3389/fpsyg.2025.1746973 (PMC12867793; doi:10.3389/fpsyg.2025.1746973)
Supplement: Supplementary file 1 [file Data_Sheet_1.docx]

Appendix A

Dear Ms./Mr.

We are conducting an academic study on *Gamified Human Resource Management and Employee Work Engagement*. Your participation will provide valuable insights into how game-based HR practices influence employee motivation and engagement. Completing this questionnaire will take approximately **8–10 minutes**.

Please note:

- Participation is voluntary.
- All responses will remain anonymous and confidential.
- The data will be used only for academic research.
- There are no risks associated with participation, and no identifiable personal data will be collected.

This study involved minimal-risk survey research and was reviewed and approved by the Academic Committee of Guangdong University of Science and Technology. No personal identifiers or sensitive information were collected.

Part I. General Information

| **Item** | **Situation** |
| --- | --- |
| 1. Gender | Male □ Female □ |
| 1. Age |  |
| 1. Education | High school □ College □ Bachelor □ Master & above □ |
| 1. Working years in current organization |  |
| 1. Job position: | Intern □ Specialist □ Supervisor □ Manager / Head □ |

Part II. Measurement Scales

1. Do you think the following statements describe your company’s HR practices?

(1 = Strongly Disagree, 5 = Strongly Agree)

| **No.** | **Item** | **Totally Disagree – Totally Agree** |
| --- | --- | --- |
| 1 | The company uses gamified mechanisms in employee training programs. | 1 2 3 4 5 |
| 2 | The organization integrates points and rewards into performance evaluation. | 1 2 3 4 5 |
| 3 | Leaderboards are applied in recruitment or assessment processes. | 1 2 3 4 5 |
| 4 | Tasks are designed as challenges to encourage participation. | 1 2 3 4 5 |
| 5 | Feedback is provided in a game-like and interactive way. | 1 2 3 4 5 |
| 6 | Gamified tools are embedded in communication between managers and employees. | 1 2 3 4 5 |
| 7 | Employee development activities include game-based elements. | 1 2 3 4 5 |

1. How well do the following statements describe your feelings about your job?

(1 = Strongly Disagree, 5 = Strongly Agree)

| **No.** | **Item** | **Totally Disagree – Totally Agree** |
| --- | --- | --- |
| 1 | At my work, I feel bursting with energy. | 1 2 3 4 5 |
| 2 | At my job, I feel strong and vigorous. | 1 2 3 4 5 |
| 3 | I am enthusiastic about my job. | 1 2 3 4 5 |
| 4 | My job inspires me. | 1 2 3 4 5 |
| 5 | I am immersed in my work. | 1 2 3 4 5 |
| 6 | I feel happy when I am working intensely. | 1 2 3 4 5 |

1. How do you feel when performing your daily work tasks?

(1 = Strongly Disagree, 5 = Strongly Agree)

| **No.** | **Item** | **Totally Disagree – Totally Agree** |
| --- | --- | --- |
| 1 | I find doing this activity interesting. | 1 2 3 4 5 |
| 2 | I enjoy the activity very much. | 1 2 3 4 5 |
| 3 | I think this task is quite fun. | 1 2 3 4 5 |
| 4 | I feel competent while doing this activity. | 1 2 3 4 5 |
| 5 | I feel pressure while doing this activity. *(Reverse-coded)* | 1 2 3 4 5 |
| 6 | I believe this activity is important for me. | 1 2 3 4 5 |

1. Please indicate the extent to which you agree with the following descriptions of your personal preferences at work.

(1 = Strongly Disagree, 5 = Strongly Agree)

| **No.** | **Item** | **Totally Disagree – Totally Agree** |
| --- | --- | --- |
| 1 | I enjoy motivating myself through competition. | 1 2 3 4 5 |
| 2 | I prefer to collaborate with others when completing tasks. | 1 2 3 4 5 |
| 3 | I feel more engaged when there is a clear ranking or leaderboard. | 1 2 3 4 5 |
| 4 | I like achieving new levels and unlocking rewards. | 1 2 3 4 5 |
| 5 | I am more motivated when I can compare my performance with others. | 1 2 3 4 5 |

1. Please indicate how much you agree with the following statements about your organization.

(1 = Strongly Disagree, 5 = Strongly Agree)

| **No.** | **Item** | **Totally Disagree – Totally Agree** |
| --- | --- | --- |
| 1 | The organization values my contributions to its well-being. | 1 2 3 4 5 |
| 2 | The organization really cares about my well-being. | 1 2 3 4 5 |
| 3 | The organization strongly considers my goals and values. | 1 2 3 4 5 |
| 4 | The organization would forgive an honest mistake. | 1 2 3 4 5 |
| 5 | The organization cares about my opinions. | 1 2 3 4 5 |
| 6 | The organization shows concern for me when I am ill. | 1 2 3 4 5 |
| 7 | The organization tries to make my job as interesting as possible. | 1 2 3 4 5 |
| 8 | The organization takes pride in my accomplishments. | 1 2 3 4 5 |

Closing Note

Thank you very much for your time and participation. Your responses are essential for advancing our understanding of gamified management and employee engagement.
